# Linking epigenetic mechanisms of T cell dysfunction with pathophysiology of type 1 diabetes mellitus

**DOI:** 10.3389/fimmu.2025.1664255

**Published:** 2025-10-29

**Authors:** Abdullah Jabri, Abdulrahman Elsalti, Mohamed Alsharif, Raghad Alsharif, Tasnim Abbad, Dania Sibai, Bader Taftafa, Abdulaziz Mhannayeh, Mohammad Imran Khan, Ahmed Yaqinuddin

**Affiliations:** ^1^ College of Medicine, Alfaisal University, Riyadh, Saudi Arabia; ^2^ International School of Medicine, Istanbul Medipol University, Istanbul, Türkiye; ^3^ College of Medicine, Almaarefa University, Riyadh, Saudi Arabia; ^4^ King Faisal Specialist Hospital and Research Center, Jeddah, Saudi Arabia

**Keywords:** type 1 diabetes, epigenetics, T-cell dysfunction, autoimmunity, DNA methyaltion

## Abstract

β-cell destruction by autoreactive T cells is a hey hallmark of type 1 diabetes mellitus (T1D). Epigenetic mechanisms—including DNA methylation, histone modifications, chromatin remodeling, and non-coding RNAs—play critical roles in regulating T-cell development, activation, and tolerance. Disruption of these processes contributes to immune imbalance and the onset of T1D. This review summarizes current insights into how epigenetic regulation shapes T-cell function and highlights emerging evidence linking these changes to environmental influences such as gut microbiota, diet, and viral infections. Exploring the interaction between genetic susceptibility and environmental triggers through an epigenetic framework not only advances our understanding of T1D pathogenesis but also provides opportunities for biomarker discovery and the development of targeted epigenetic therapies. With further research, these advances hold promise for improving precision medicine strategies in T1D.

## 1 Introduction

Type 1 diabetes (T1D) is an autoimmune disease in which the immune system mistakenly attacks and destroys pancreatic β-cells, eventually leading to insulin shortage (1). This β-cell loss primarily happens via autoreactive T-cell mechanisms in genetically predisposed individuals, often initiated or modulated by environmental factors (2). Epigenetic regulation—through mechanisms such as DNA methylation, histone modifications, non-coding RNAs, and chromatin remodeling—has emerged as a critical mediator linking genetic susceptibility to environmental influences in T1D (3). These heritable yet reversible modifications govern T-cell development, activation, and tolerance, thereby shaping immune balance and disease risk (4).

The disease progresses through three clinically and biologically distinct, largely silent stages (5, 6). In Stage 1, individuals have detectable autoantibodies directed against pancreatic β-cell antigens, indicating an active immune attack on the islets, but glucose metabolism remains within the normal range and there are no symptoms. In Stage 2, ongoing immune-mediated β-cell injury produces measurable impairment of glucose regulation, for example abnormal responses on glucose tolerance testing or a rising A1c although fasting glucose and symptom status may still be non-diabetic. In Stage 3, β-cell loss reaches a threshold at which persistent hyperglycemia develops, meeting diagnostic criteria for diabetes and often accompanied by typical symptoms such as polyuria, polydipsia, and weight loss. CD4^+^ and CD8^+^ T-cells are essential factors in the progression of T1D and significant elements of the islet infiltration. Initially, autoreactive T cells are stimulated by β-cell antigens shown by antigen-presenting cells (APCs) (7). The activated CD4^+^ T-cells invade the pancreas and are believed to aid in β-cell damage through the activation of macrophages and CD8^+^ T-cells. These in turn are directly responsible for the destruction of β-cells through their interaction with major histocompatibility complex (MHC) class I molecules and by the secretion of perforin and granzyme (8). Usually, regulatory T cells (Tregs), the main regulators of inflammatory responses, are responsible for immune tolerance and homeostasis (9, 10). The lack of Tregs may become one of the reasons for the development of human autoimmune diseases like T1D, whereas an excess of Tregs may lead to the weakening of the immune response to cancer or infections (11). Established T1D risk genes include the human leukocyte antigen (HLA) region, insulin (INS), protein tyrosine phosphatase non-receptor type 22 (PTPN22), interleukin-2 receptor alpha (IL2RA), and cytotoxic T-lymphocyte associated protein 4 (CTLA4), among others (12). Importantly, their expression is strongly influenced by epigenetic mechanisms, which may explain how environmental exposures—such as viral infections, microbiota alterations, or dietary factors—trigger or accelerate disease onset. In this review, we aimed to explore the role of epigenetic regulation of T cells in the pathogenesis of T1D, with a particular focus on how mechanisms such as DNA methylation, histone modifications, non-coding RNAs, and chromatin remodeling influence T-cell development, activation, and tolerance. By summarizing current findings on epigenetic dysregulation in both CD4^+^ and CD8^+^ T-cell subsets, and examining the interplay between environmental triggers and genetic susceptibility, we highlight the growing importance of epigenetic biomarkers for diagnosis and the therapeutic potential of epigenome-targeting strategies.

## 2 Overview of epigenetic modifications

### 2.1 DNA methylation: a versatile and targeted regulator

DNA methylation is a key regulatory process of the addition of a methyl group to cytosine bases within Cytosine-phosphate-Guanine (CpG) dinucleotides and is mediated by DNA methyltransferases (DNMTs). In general, promoter methylation is silencing and demethylation is activation (13). For example, the transcription factor FOXP3, essential for Treg development, is silenced when its regulatory regions are hypermethylated and activated when hypomethylated (14). This helps to maintain immune tolerance and Treg lineage fidelity.

Region specific hypomethylation also activates immune related genes. Genes such as HLA-DQB1 and GAD2 have lower methylation at their promoters and enhancers under immunostimulatory conditions which in turn enhance antigen presentation and cytokine responsiveness (15). These are not stochastic but occur at defined regulatory loci, so it’s a tightly controlled system of gene activation and silencing.

Methylation variability refers to the differences in DNA methylation patterns observed across individuals, tissues, developmental stages, or environmental conditions (16). It has been noticed even between genetically identical monozygotic twins. High resolution methylome studies show differential methylation at loci including INS-IGF2, SH2B3 and MEG3 (17). This inter-individual variation gives insight into how genetically similar individuals can have different immunological outcomes. Abnormal methylation variability is often associated with pathological conditions, including cancer, autoimmune disorders, and neurological diseases, making it a valuable biomarker for disease risk and progression.

### 2.2 Histone modifications: balancing activation and Repression

Histone modifications are post-translational changes to the histone tails that wrap DNA into chromatin (15). These changes control chromatin accessibility and help recruit transcription factors. For example, H3K9 acetylation (H3KAc) is associated with open chromatin and active transcription, especially at immune genes like HLA-DRB1/DQB1 in APCs (13). H3K9 demethylation (H3K9me2) at loci like CTLA4 is linked to a repressive chromatin state, preserving immune checkpoints and preventing autoreactivity.

Histone acetylation patterns are sensitive to environmental cues. Microbial metabolites like butyrate, a short chain fatty acid produced by commensal Clostridium species (clusters IV/XIVa) inhibit histone deacetylases (HDACs) and promote acetylation at immune regulatory loci like FOXP3 (18). This leads to enhanced Treg differentiation and immune homeostasis. Epigenetic integration of microbiota derived signals is key to immune tolerance.

Metabolic factors also affect histone modification patterns. For example, hyperglycemia decreases the activity of NAD^+^-dependent deacetylases SIRT2 and SIRT6, leading to persistent acetylation at histone residues H3K9, H3K14 and H3K27 (19). These modifications impair β-cell function, alter stress response gene expression and may contribute to long term metabolic complications. Histone modifications are epigenetic sensors of both microbial and metabolic environments.

### 2.3 Non-coding RNAs: epigenetic regulators in health and disease

Both post-transcriptional and chromatin levels of gene expression are controlled by non-coding RNAs (ncRNAs), which include microRNAs (miRNAs) and long non-coding RNAs (lncRNAs) (20). miRNAs usually attach to the 3′ untranslated regions of target mRNAs to prevent translation or degradation. Treg migration, cytokine signaling and immunological homeostasis are controlled by miRNAs like miR-125a-5p and miR-342 in immune cells. Changes in immune response and disease susceptibility are linked to polymorphisms in regulatory miRNAs like miR-146a and miR-155 (15).

Regulatory miRNAs like miR-375 are present in pancreatic β-cells where they have a role in insulin secretion and β-cell survival. miR-375 is upregulated in normal conditions resulting in the suppression of insulin secretion by targeting exocytosis-related genes (e.g. Myotrophin, PDK1), but chronic high glucose downregulates miR-375 and leads to dysregulated insulin release and β-cell stress (13, 15, 21). miRNAs maintain endocrine cell identity while fine-tuning immunological responses.

lncRNAs are more than 200 nucleotides long and act through various mechanisms, including chromatin looping, transcriptional interference and enhancer modulation. HI-LNC25 (LINC01370) regulates the transcription factor GLIS3 which is critical for β-cell survival and differentiation, while PLUTO promotes the expression of PDX1 a master regulator of insulin production (15). These lncRNAs have been shown to be tissue-specific epigenetic regulators. Importantly, recent evidence suggests that lncRNAs may contribute to disease susceptibility by interacting with non-coding genomic regions. More than 90% of T1D-associated single nucleotide polymorphism (SNP) are in non-coding regions, and lncRNAs are implicated in the development of autoimmune risk. One example is the SNP of NONHSAG044354 lncRNA within the BACH2 locus, a gene involved in immunoregulation and tolerance (15, 22). lncRNAs also maintain epigenetic memory by stabilizing transcriptional activity at inflammatory loci even after cytokine signaling has ceased, and thus preserve cellular identity over time (21).

### 2.4 Chromatin remodeling: organizing the accessible genome

Chromatin remodeling is the repositioning of nucleosomes by ATP-dependent complexes like SWI/SNF which control DNA accessibility to transcription factors (23). This is important during T-cell lineage differentiation, β-cell specification and enhancer activation (14).

Recent single-nucleus assay for transposase-accessible chromatin using sequencing (snATAC-seq) studies on over 130,000 nuclei have shown that many autoimmune risk variants map to cis-regulatory elements (cCREs) in memory CD8+ T cells and Tregs (23). These elements are required for gene accessibility of CTLA4 and FOXP3 which are central to immune regulation (22). Chromatin accessibility at these sites is controlled by transcription factor binding and is disrupted by disease associated variants.

Genome organizers such as Special AT-rich Sequence-Binding Protein 1 (SATB1) control long range enhancer-promoter interactions to shape chromatin (14). SATB1 promotes thymic growth and peripheral function in Tregs by opening chromatin at super-enhancers near FOXP3 and CTLA4 (24). Regulatory programs during T cell activation and differentiation rely on these remodeling activities. Inflammatory cytokines like IL-1β and IFN-γ also dynamically control chromatin accessibility. These signals open up closed chromatin regions enriched for IRF, STAT and NF-κB motifs (21, 22). This plasticity allows for rapid transcriptional responses in immune and endocrine cells. Furthermore, HLA class II haplotypes, DR3/DQ2 regulate allele specific chromatin remodeling (25). For example they control HLA-DRB5 in dendritic cells and immunological tolerance and antigen presentation.

## 3 Epigenetic dysregulation in T1D-associated T-cells

### 3.1 CD4+ T-Cells (Th1, Th17, Tregs)

Tregs in T1D undergo epigenetic changes that can disable them. Alterations in FOXP3 methylation have been reported in subsets of autoimmune diabetes. Examples include FOXP3 promoter/Treg-specific demethylated region (TSDR) hypermethylation and reduced FOXP3 expression in CD4^+^ T cells from Latent Autoimmune Diabetes in Adults (LADA) and fulminant T1D patients, and enrichment of TSDR-methylated FOXP3^+^IFN-γ^+^ cells in T1D cohorts (26–28). This epigenetic silencing can be exacerbated by environmental factors; reduced butyrate from gut dysbiosis decreases histone acetylation at the *FOXP3* enhancer and further destabilizes Treg function (18). *IL2RA* (CD25) promoter hypermethylation limits IL-2 signaling, necessary for Treg survival and suppressive capacity (29). These changes present early in disease progression, thus may contribute to breakdown of immune tolerance before clinical onset (17).

Unlike Treg dysfunction, effector CD4+ subsets (Th1 and Th17 cells) in T1D display activating epigenetic modifications at pro-inflammatory cytokine loci. Studies of Th1/Th17 lineage-specific chromatin have shown that enhancers of cytokine genes such as IFN-γ and IL-17 are marked by activating histone modifications, including H3K27ac, which facilitates transcriptional upregulation (22, 30). Moreover, single-cell chromatin accessibility analyses suggest that T1D risk variants are enriched in Th1/Th17-specific regulatory elements, potentially altering transcription factor binding and cytokine expression (23).

**Figure 1:**
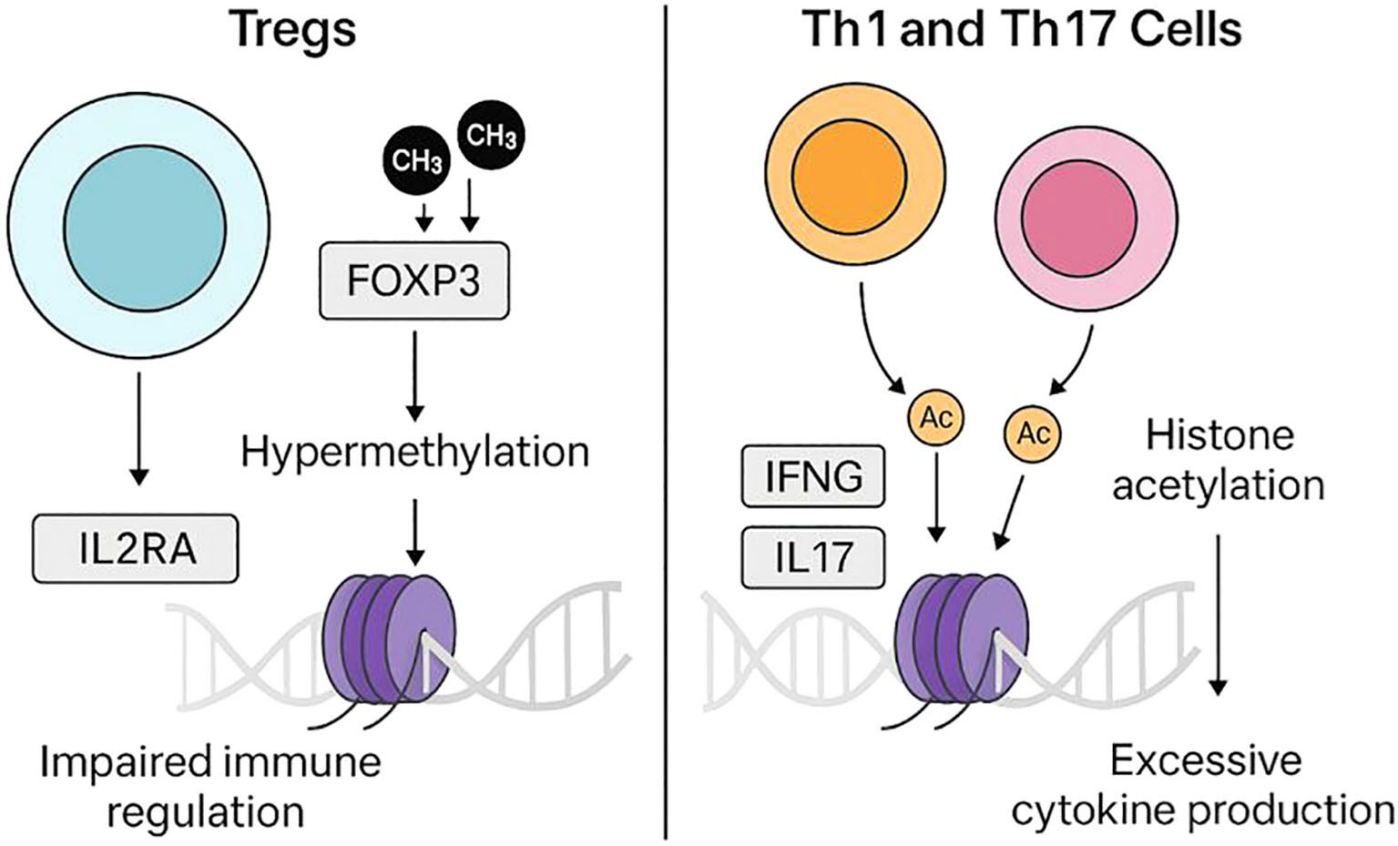
Epigenetic dysregulation of T-cell subsets in T1D. On the left, regulatory T cells (Tregs) exhibit altered epigenetic regulation at immune tolerance–related loci, including FOXP3 and IL2RA, which may impair their suppressive function. On the right, effector T cells (Th1 and Th17) display activating histone acetylation (e.g., H3K27ac) at cytokine gene loci (*IFNG*, *IL17A*), increasing pro-inflammatory cytokine production. Together, these opposing epigenetic changes weaken tolerance and promote autoimmunity in T1D.

It remains unclear whether Th1 and Th17 markers arise from the same cells (reflecting cellular plasticity) or from distinct subsets, as studies report both scenarios (31). Similarly, checkpoint receptor changes such as CTLA4 and PD-1 may not be uniform across all T1D patients; some studies report reduced PD-1 expression on Tregs, whereas others find normal levels. Frequencies and suppressive function of Tregs also show conflicting results across cohorts. Comparative commentary indicates that while some studies report increased Th17 cells in T1D, others find no change in IL-17–producing cells under baseline conditions. These inconsistencies underscore heterogeneity among patients and highlight the need for careful interpretation of immune signatures. Together, Treg dysfunction and Th1/Th17 hyperactivity may create a self-reinforcing cycle of autoimmunity (**Figure 1**).

### 3.2 CD8+ T-cells (cytotoxic T-cells)

CD8+ T-cells are primed for autoreactivity in T1D through epigenetic changes that make them more reactive to β-cell antigens. Epigenetic variation in immune cells, such as altered methylation at loci including INS and IL2RA, has been associated with T1D risk. In parallel, molecular mimicry between β-cell autoantigens (e.g., insulin, GAD65) and microbial peptides may promote activation of autoreactive T cells (16, 18). Enhancers near genes involved in cytotoxic function, such as SOCS1 (cytokine signaling) and STXBP1 (vesicle fusion) are commonly disrupted in T1D patients according to chromatin accessibility profiling (30). CD8+ T-cells are more cytotoxic in T1D patients due to these epigenetic changes.

The T1D microenvironment activates CD8+ T-cells. IFN-γ induces MHC class I on β-cells, making them more visible to cytotoxic T-cells (32). miR-23b, miR-590-5p dysregulate CD8+ T-cell survival by suppressing TRAIL and FAS (33). Notably, these epigenetic changes occur early in disease progression, as seen by hypomethylation at the LDHC locus in children who later develop autoantibodies (25). Thus, these mechanisms create a self-reinforcing cycle where epigenetic priming activates CD8+ T-cells, which in turn destroy more β-cells and release more antigen.

### 3.3 Dysregulation of immune tolerance

One mechanism of tolerance breakdown in T1D is epigenetic silencing of immunological checkpoint molecules. T1D patients have hypermethylation at the CTLA4 and PD-1 loci which reduces expression of these inhibitory receptors (30, 34). Variants in the CTLA4 enhancer region can make this worse by disrupting chromatin architecture and transcription factor binding (IRF1) in Tregs (24). These epigenetic changes lead to autoimmune β-cell death,compromised checkpoint function and uncontrolled T cell activation. **Table 1**. summarizes several epigenetic changes in association with T1D.

**Table 1:** Contrasting epigenetic regulation of CD4^+^ vs CD8^+^ T cells in T1D.

Feature	CD4^+^ T cells (Tregs, Th1, Th17)	CD8^+^ T cells (Cytotoxic)
Primary epigenetic change	FOXP3 and IL2RA hypermethylation (↓ tolerance); H3K27ac at IFNG/IL17 loci (↑ cytokine activity)	Hypomethylation at INS and GAD65 (↑ antigen recognition); enhancer disruption at SOCS1, STXBP1 (↑ cytotoxicity)
Functional outcome	Loss of immune regulation (weakened Treg suppression) and overproduction of pro-inflammatory cytokines	Increased autoreactivity, survival, and cytotoxic potential of CD8^+^ T cells
Environmental triggers	Gut dysbiosis (↓ butyrate → FOXP3 silencing); viral infections (↑ histone acetylation at cytokine loci)	Molecular mimicry (Bacteroides); metabolic stress/high-fat diet; IFN-γ induction of β-cell MHC-I
Key consequence	Breakdown of tolerance → failure to restrain autoimmunity	Direct β-cell destruction → amplification of antigen release and immune activation
Pathogenic role in T1D	“Gatekeepers” of tolerance fail, allowing autoreactive responses to persist	“Executioners” of β-cell damage, driving irreversible β-cell loss

### 3.4 Long non-coding RNAs in T-cell regulation

lncRNAs shape T-cell fate and effector programs by scaffolding chromatin modifiers, guiding transcription-factor recruitment, and modulating enhancer–promoter communication. In Tregs, Flicr (Foxp3 long intergenic non-coding RNA) acts as a negative tuner of FOXP3, altering chromatin accessibility at Foxp3 regulatory elements; genetic ablation increases FOXP3 and improves tolerance in autoimmune-prone backgrounds, highlighting Flicr as a rheostat of Treg stability (35).

In Th1 cells, the antisense lncRNA NeST (also known as Tmevpg1/Ifng-AS1) is induced in a T-bet/STAT4–dependent manner and promotes IFNG transcription by recruiting WDR5/MLL to deposit H3K4 methylation at the Ifng locus; NeST thus reinforces Th1 polarization and IFN-γ output (36–38).

For Th2 programs, lincR-Ccr2-5′AS cooperates with GATA-3 to regulate a chemokine-receptor cluster (CCR1/2/3/5), and its knockdown impairs Th2 migration *in vivo*, illustrating how lncRNAs coordinate lineage-specific trafficking with gene programs (39).

## 4 Gene–environment–epigenome interactions

### 4.1 Genetic susceptibility

T1D susceptibility is strongly influenced by genetics, with 78 risk loci now identified by large GWAS and fine-mapping studies (23, 40–52). Many of these risk variants fall in regulatory elements active in immune and pancreatic cell types, suggesting functional effects on gene expression (23). Recent research indicates that epigenetic mechanisms may be involved in the development of T1D due to genetic risk variations. SNPs in INS (rs689) and IL2RA (rs12722495) in particular were linked to altered DNA methylation at immune cell promoter CpG sites. Higher methylation in CD8^+^ T cells was associated with the risk allele rs689, but lower methylation was found in B cells with rs12722495. These methylation alterations specific to a genotype may affect immunological functioning and increase the risk of developing the disease (53). **Table 2** summarizes key genes and their associated SNPs linked to immune function, β-cell regulation, and T1D risk (49). However, many of these genes, such as CCR5, IL10, IL27, and GPX7, have been variably reported in association with T1D. For instance, CCR5 has shown associations in some populations but not others, and IL10/IL27 findings have been inconsistent across cohorts. These discrepancies suggest that some of these loci may have modest effect sizes or population-specific effects. Therefore, while these genes are candidates for contributing to T1D susceptibility, many associations remain tentative, and their precise functional roles in disease pathogenesis are still uncertain.

**Table 2:** Summary of key genes and their associated SNPs linked to immune function, β-cell regulation, and T1D risk.

Gene/region	SNP	Function	Reference(s)
HLA Class II	rs6927022, rs2157051, rs9275184, rs7744001	Presents antigens to CD4+ T-cells for immune recognition	(157)
CTLA4	rs11571316, rs3087243	Immune checkpoint protein that suppresses T-cell activation	(55, 158)
CCR5	rs113010081	Affects immune cell function and signaling	(51)
TLR7/8	rs5979785	Detects pathogens and triggers immune responses	(159)
AFF3	rs9653442	Regulates gene transcription; linked to immune cell and cancer development	(160)
INS	rs7111341	Encodes insulin, lowering blood glucose levels	(43)
GLIS3	rs7020673, rs10758593	Supports pancreatic β-cell development and insulin pr	(42)
BAD	rs694739	Promotes programmed cell death (apoptosis)	(161)
IL7R	rs11954020	Facilitates immune responses, antibody production, and T-cell cytotoxicity	(51)
IL10	rs3024505	Suppresses inflammation (anti-inflammatory cytokine)	(158)
IL27	rs151234	Modulates T-cell activity and inhibits excessive proliferation	(51)
WFS1	rs1046322	Protects β-cells and brain cells from stress in the endoplasmic reticulum	(162, 163)
CTSB	rs1296023	Breaks down proteins in lysosomes	(161)
CTSH	rs3825932	Essential for lysosomal protein degradation	(158)
GPX7	–	Regulates pancreatic β-cell growth and survival	(132)
GSTT1	–	Influences β-cell proliferation and death	(132)
SNX19	–	Plays a role in β-cell maintenance and apoptosis	(132)

Adapted from: Mittal et al. “Gene-environment interaction in the pathophysiology (59).

#### 4.1.1 Human leukocyte antigen

About 50% of the lifetime risk of T1D is attributed to mutations in the HLA class II genes on chromosome 6, which increases the chance of acquiring the disease (47, 54). Specifically, the DR4-DQ8 (DQA1*03:01 – DQB1*03:02) or DR3-DQ2 (DQA1*05:01 – DQB1*02:01) haplotypes are present in 90% of children with T1D. The largest risk factor for contracting the disease is the combination of these two haplotypes in a person’s genotype (55). Numerous studies have examined the connection between T1D risk and variations in the HLA gene. These genetic correlations have implications for disease prediction and means of prevention in addition to aiding in our understanding of the pathophysiology of T1D. HLA typing, for instance, is utilized in T1D prevention trials to identify people who might benefit from early interventions and to stratify risk (56).

#### 4.1.2 Cathepsin H

Other gene loci, including the susceptibility locus of cathepsin H (CTSH), have also been linked to the development of T1D in addition to HLA. CTSH has been linked to a higher incidence of T1D by genome-wide association studies (GWAS) (51). Using integrated data from quantitative trait locus (eQTL) with GWAS, a study identified the possible pathogenic pathways of the CTSH gene in T1D (57). Single cell RNA sequencing (scRNA) revealed that the pancreas of T1D patients had a significant upregulation of the CTSH gene in acinar cells as compared to the control group. Additionally, a group of genes co-expressed with CTSH that had a substantial positive connection with T1D were found using single-cell weighted gene co-expression network analysis (WGCNA). The CTSH gene in the exocrine pancreas was thought to enhance the antiviral response based on functional enrichment analysis. An inflammatory milieu is produced as a result of this amplification, which also raises the expression of pro-inflammatory cytokines. T1D is likely to develop as a result of this process, which is likely to harm β-cells. High CTSH expression, which is influenced by other environmental factors such post-translational modifications and epigenetics, was found to connect with the risk of T1D in another study (58). When combined, these studies demonstrate how CTSH contributes to a higher risk of T1D development.

#### 4.1.3 Other genes

It has been demonstrated that additional potential genes, including INS, GLIS3, CCR5, BAD, GPX7, GSTT1, and SNX19, increase vulnerability to T1D (23, 40–51). A few of these genes have a direct impact on pancreatic β-cell growth and death. **Table 3** provides a detailed list of all the genes linked to a higher risk of T1D along with an explanation of their roles.

**Table 3:** Epigenetic dysregulation in T1D-associated T-cells.

Cell type	Epigenetic change	Affected gene/pathway	Environmental trigger	Mechanism	Evidence source	Reference(s)
Tregs	*FOXP3* hypermethylation	↓ Treg function	Gut dysbiosis (↓ butyrate)	↓ Histone acetylation → *FOXP3* silencing	Human, *in vitro*	(13, 20)
	*IL2RA* hypermethylation	↓ IL-2 signaling	—	Promoter methylation → ↓ IL-2 responsiveness	Human, animal	(14, 24)
Th1/Th17	*IFN-γ/*IL-17 H3K27ac	↑ Pro-inflammatory	Viral infections (coxsackievirus)	Histone acetylation → cytokine overproduction	Human, animal, *in vitro*	(22, 30)
CD8+ T-cells	*INS*/*GAD65* hypomethylation	↑ Autoantigen reactivity	Molecular mimicry (*Bacteroides*)	Hypomethylation → ↑ autoantigen recognition	Human	(16, 18)
	*LDHC* hypomethylation	Early priming	Diet (high-fat)	Metabolic stress → epigenetic priming	Human cohort, animal	(25, 33)
	miR-23b downregulation	↑ TRAIL/FAS signaling	—	Dysregulated miRNAs → ↑ CD8+ T-cell survival	Animal, *in vitro*	(33)
Immune Checkpoints	CTLA-4 hypermethylation	↓ Treg suppression	—	Promoter methylation → ↓ checkpoint inhibition	Human	(30, 34)
Systemic	Genome-wide hypomethylation	↑ Autoimmunity	Enterovirus infection	IFN-α → ↓ DNMT activity → global hypomethylation	Human pancreas, animal	(21)
	TLR4/NF-κB activation	↑ Pro-inflammatory	LPS (Gram-negative bacteria)	Microbiota-derived LPS → TLR4 signaling → inflammation	Human (children), animal	(164)

Recent research have demonstrated that the pathophysiology of T1D is complex, despite the fact that genetics has been found to play a significant influence in the disease. Identical twin studies have revealed that if one twin has T1D, the other twin may not be at all susceptible to the condition, indicating that genetic factors by themselves are insufficient to fully explain how T1D develops (59).

### 4.2 Environmental triggers

In addition to genetics, environmental factors have been linked to the development of T1D independently. These include viral infections, pesticide exposure, lifestyle and eating habits, and vitamin D deficiency (60–62).

#### 4.2.1 Viral infections

**Figure 2:**
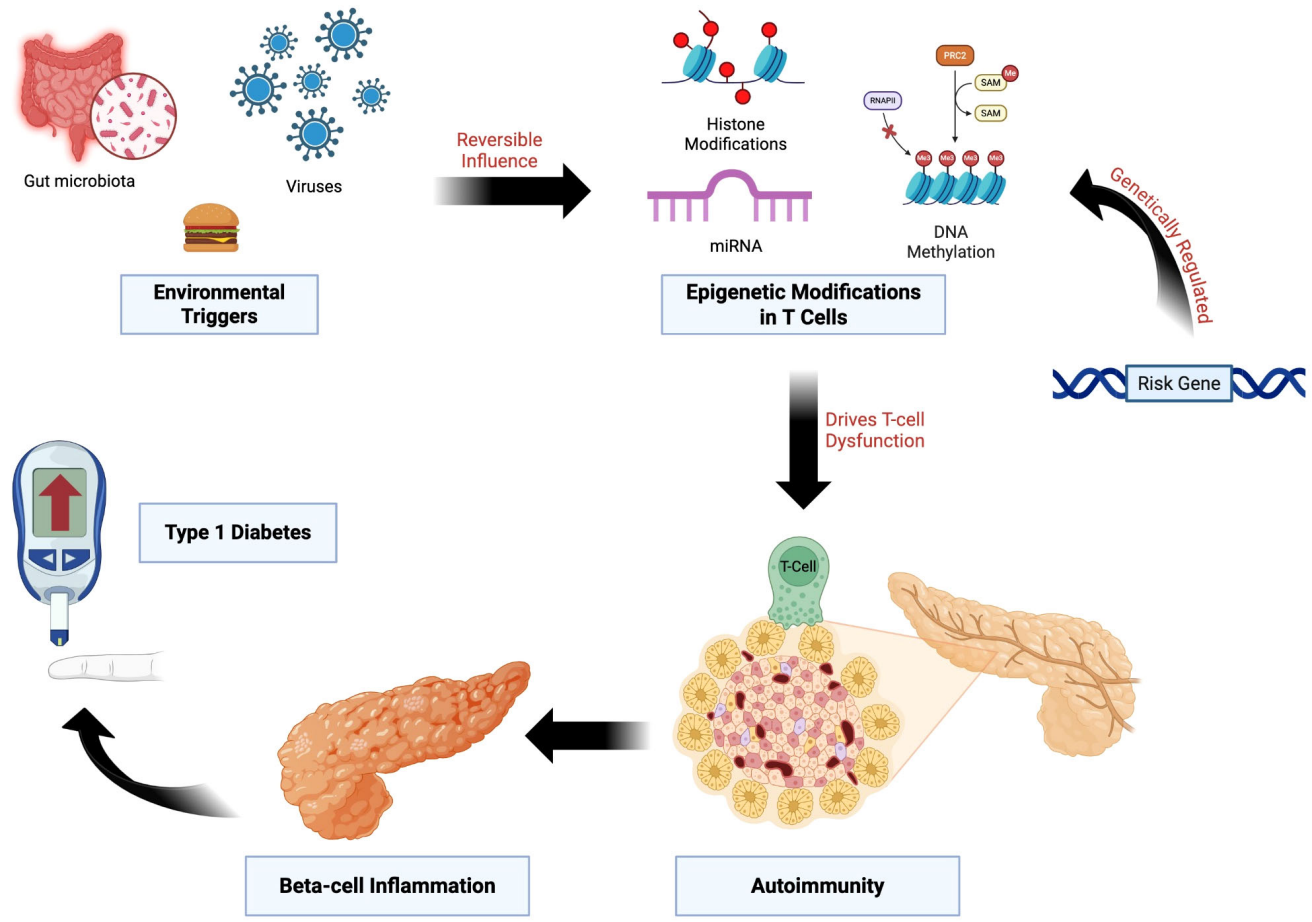
Epigenetic Dysregulation Linking Genetic Risk and Environmental Triggers to T1D. Genetic susceptibility loci influence epigenetic regulation in T cells through mechanisms including DNA methylation, histone modifications, and non-coding RNAs. Environmental exposures, such as viral infections, altered gut microbiota, and dietary factors, exert reversible effects on these pathways. The resulting epigenetic dysregulation promotes loss of immune tolerance, β-cell inflammation, and progression to T1D.

Viral infection-induced autoimmunity may be a significant factor in the development of T1D (**Figure 2**) (63). Enteroviruses have been linked to the etiopathogenesis of T1D on several levels, including infecting pancreatic β-cells and triggering autoimmunity against them (64). Most commonly, T1D incidence has been linked to Coxsackie B viruses (65–67). Enterovirus proteins have been detected in the pancreas during the outset of illness in people with T1D (68). It has been demonstrated that several enterovirus species can infect and impair the function of pancreatic β-cells since these cells also contain many receptors that enteroviruses employ to entry into cells. Interferons, which are produced in response to these viral infections, drive gene transcription; newly diagnosed T1D patients have been found to exhibit this IFN-stimulated gene expression. The later emergence of autoantibodies against pancreatic β-cells has also been linked to this gene transcription. Given that viremia was missing in children with quick onset T1D in the TEDDY research, it is possible that infections could cause autoimmunity gradually over time as opposed to suddenly (69). Moreover, pancreatic β-cell antigens and certain viruses, like enteroviruses, have structural similarities. This similarity may result in a condition called molecular mimicry, in which the body’s own cells, including β-cells that produce insulin, are mistakenly attacked by the immune system, which is triggered to combat the virus, causing T1D (60). In pancreatic β-cells, enteroviruses have been demonstrated to interfere with the miRNA-mediated inhibition of pro-inflammatory pathways, whereas related Picornaviridae viruses, like rhinovirus, can modify the expression of cytokine genes by altering DNA methylation (70–73). The offspring may be primed for autoimmune reactions and have a higher chance of developing T1D later in life if the mother’s enteroviral infection during pregnancy causes long-lasting epigenetic changes in the fetal immune-related genes (74–77).

#### 4.2.2 Pesticide exposure

T1D development has been linked to pesticide exposure. Chemicals called pesticides are used extensively in agriculture to control pests, but there have been worries about their possible effects on human health. Studies have examined the link between pesticide exposure and T1D, although research in this field is still ongoing and results are inconclusive (78). Epidemiological studies suggest that environmental toxins such as pesticides may interact with genetic susceptibility to influence disease onset. There may be a connection between pesticide exposure and T1D, according to epidemiological research. Even at low concentrations, pesticide exposure has been linked to the occurrence of T1D and prediabetes, also known as aberrant glucose regulation (79). Men and women had different causal relationships between pesticide exposure and impaired glucose control; in men, a U-shaped dose-response relationship was more pronounced.

Pesticides may also cause or hasten the autoimmune reaction that destroys β-cells in the pancreas, according to some theories. Although the exact processes behind this possible link are unknown, they might have to do with oxidative stress induction or immune function disturbance (59). These findings illustrate how environmental exposures beyond viral infections can contribute to T1D development, setting the stage to examine lifestyle and dietary influences.

#### 4.2.3 Lifestyle and diet

Numerous studies have examined the relationship between dietary and lifestyle factors and the onset of T1D, identifying a number of connections and possible mechanisms (80). Diet and lifestyle represent modifiable environmental factors that may mediate T1D risk, in part through their effects on gut microbiota and immune function. It is clear that dietary practices that alter the composition of the gut microbiota may be a major factor in the development of T1D. Up to now, the most convincing evidence for a causal link between intestinal microbiome and the disease comes from well-controlled intervention studies in murine models (81). Although not fully understood, a complicated relationship between gut permeability, the immune system, and intestinal microbiota has previously been discovered (82). The gut barrier, which is made up of enterocytes, mucus, gut microbiota, tight junction (TJ) proteins, and the innate and adaptive immune cells that make up the gut-associated lymphoid tissue, regulates gut permeability (83). Intestinal permeability and the passage of microbial antigens, products, or microbes themselves can result from the breakdown of TJ and the compromise of the intestinal barrier. The expression of TJ proteins, which include claudin-2, occludin, cingulin, and zonula occludens (ZO) proteins, controls the TJ of the intestinal barrier. According to some research, intestinal permeability is dependent on elevated zonulin levels, which are impacted by bacterial colonization (84, 85). It is also known that zonulin modulates TJ to reversibly modify intestinal permeability (86–88). It is interesting to note that elevated blood zonulin levels occur prior to the development of clinically noticeable T1D (89). However, subsequent studies have raised concerns regarding the specificity of zonulin assays and the generalizability of these findings. Critical reviews indicate that while zonulin represents a potentially important modulator of intestinal permeability, its measurement can be affected by cross-reactivity and methodological variability, and not all individuals with T1D show elevated levels. Therefore, interpreting zonulin data requires caution, and it should be considered alongside other markers and functional assessments of intestinal barrier integrity. Furthermore, an increase in intestinal paracellular permeability has been found in T1D patients, supporting the concept of barrier dysfunction as a feature of disease pathogenesis (90–93).

Intestinal permeability was higher in children with multiple islet autoantibodies (≥2 IA) who developed T1D than in those who did not, indicating a role for intestinal permeability in the pathophysiology of T1D (94, 95). The intestinal barrier’s permeability is modulated by a variety of gut commensals (96). The data that certain gut bacteria create gamma-aminobutyric acid and express GAD supports a theory. By acting as an antigen to activate submucosal T-cells, the GAD produced from bacteria as a result of gut bacterial death (e.g., by viral or antibiotic-mediated mechanisms) may miseducate the host immune system and result in the development of T1D (97, 98).

Some of the bacteria can carry peptide sequences that resemble insulin, which could cause auto-immunity, according to bioinformatics research (99). Remarkably, T-cell clones that are directed against preproinsulin peptides have demonstrated a high degree of cross-reactivity with peptides from Clostridium and Bacteroides species (100). A peptide generated by Parabacteroides distasonis that resembles the β-chain of insulin has been found in a NOD mouse model (101). T-cells are able to identify this peptide, which triggers an immunological reaction to this insulin chain.

The gnotobiotic zebrafish model has shown that the intestinal microbiota is necessary for the normal growth of the pancreatic β-cell population during early larval development. This is due to the action of a bacterial protein called β-cell expansion factor A (BefA), which is produced by gut microbes (102). These results raise the possibility that the gut microbiota plays a part in the formation of early pancreatic β-cells and point to a connection between juvenile fecal microbiota composition and an elevated risk of diabetes.

Studies have repeatedly shown that T1D is linked to notable changes in the makeup of the gut microbiota. In comparison to healthy controls, children who subsequently developed T1D had different microbial patterns, including lower levels of Lactococcus lactis and Streptococcus thermophilus and greater levels of Bifidobacterium spp., according to the seminal TEDDY study (103). Bacteroides species are more prevalent in both established T1D patients and at-risk individuals, according to several independent studies (104–106). Certain strains, such as B. dorei and B. vulgatus, are particularly enriched in high-risk Finnish children (107), while B. stercoris, B. intestinalis, B. cellulosilyticus, and B. fragilis are found in Italian patients (108).

However, results across cohorts have not always been consistent, indicating that microbial signatures of T1D risk are still unclear. For example, while the TEDDY longitudinal analysis found differences in early microbiota, other cohorts such as Diabimmune and DIPP report different taxa changes (103). Recent reviews and meta-analyses emphasize that microbial findings vary by geography and study population, and some studies fail to replicate specific “diabetogenic” bacteria (109).

Two trends that stand out in functional investigations of the gut microbiome in T1D are the significant drop in butyrate-producing bacteria from Clostridium clusters IV and XIVa and the decreased number of species that break down mucin, such as Prevotella and Akkermansia (110, 111). Studies using metagenomic and metabolomic techniques have found common microbial traits between T1D patients and their siblings, such as higher Clostridiales and Dorea with concomitant reductions in Dialister and Akkermansia (111) These alterations seem to be clinically significant.

One especially noteworthy observation is the reduction of butyrate-producing bacteria, which has been linked in several studies to greater intestinal permeability and an increased risk of T1D (110, 112–114). Intervention studies that demonstrate that butyrate supplementation can enhance metabolic parameters and cause disease remission in NOD mice models further reinforce this relationship (115, 116). The exact molecular pathways are still unclear, highlighting a crucial field for further investigation, even though these findings collectively strongly link gut microbiota dysbiosis to T1D development, especially through processes involving barrier function and immune modulation.

Systemic immunological responses are significantly shaped by short-chain fatty acids (SCFAs) generated from the microbiota, especially butyrate and propionate (117). The pancreas and lymph nodes are among the distal tissues that these compounds affect after diffusing through the intestinal epithelium. Through processes including HDACs inhibition and free fatty acid receptor (FFAR) activation, SCFAs control T-cell activity. This results in epigenetic remodeling that promotes the formation of regulatory T cells and lowers inflammation. However, host-specific variables including nutrition, metabolic status, and microbiome makeup affect SCFA effects, which are highly context-dependent. In order to completely comprehend and utilize SCFA-driven immune regulation in the setting of T1D, it may be necessary to integrate metagenomic, metabolomic, and epigenetic techniques.

Vitamin D represents another dietary factor that may influence T1D risk through immune modulation. The onset of T1D has been linked to low vitamin D levels (118–121). This correlation is believed to result from vitamin D’s possible ability to influence immune system modulation, which may have an effect on the autoimmune processes implicated in T1D. Other research, however, has found no link between low vitamin D levels and increased incidence of T1D (122, 123). Furthermore, interventional studies investigating vitamin D supplementation for T1D prevention have produced mixed results. For example, a 2021 meta-analysis reported limited and inconsistent evidence that vitamin D supplementation reduces T1D risk, highlighting variability in study design, population, and dosing regimens. These findings indicate that, while vitamin D may have immunomodulatory effects, supplementation alone has not been conclusively shown to prevent T1D (124).

Vitamin D affects immune function through genetic pathways involving the vitamin D receptor (VDR), in addition to its traditional role in maintaining mineral homeostasis (125). VDR binds to vitamin D response elements (VDREs) and forms a heterodimer with retinoid X receptor (RXR) upon binding 1,25(OH)D (126). This heterodimer regulates transcription differently depending on the cell type. Vitamin D’s specific immunomodulatory effects on T cells and other immunological subsets implicated in T1D may be explained by this. Furthermore, complexes of vitamin D and VDR can disrupt transcription factors like CREB, altering gene expression without the involvement of RXR and pointing to different epigenetic processes of immune control (112).

#### 4.2.4 Antibiotic use

Research has indicated a link between the use of antibiotics and a higher risk of developing T1D (80, 127, 128). Depending on the route of delivery, using broad-spectrum antibiotics during the first two years of life has been linked to an increased risk of developing T1D (129). Interestingly, only infants born via cesarean section showed a correlation between broad-spectrum antibiotics and T1D, but kids born vaginally did not. Other research, however, finds no connection between T1D and antibiotic use (130, 131). To determine the role of antibiotic use and delivery method in the development of T1D, more research is necessary.

### 4.3 Integrated mechanisms

**Figure 3:**
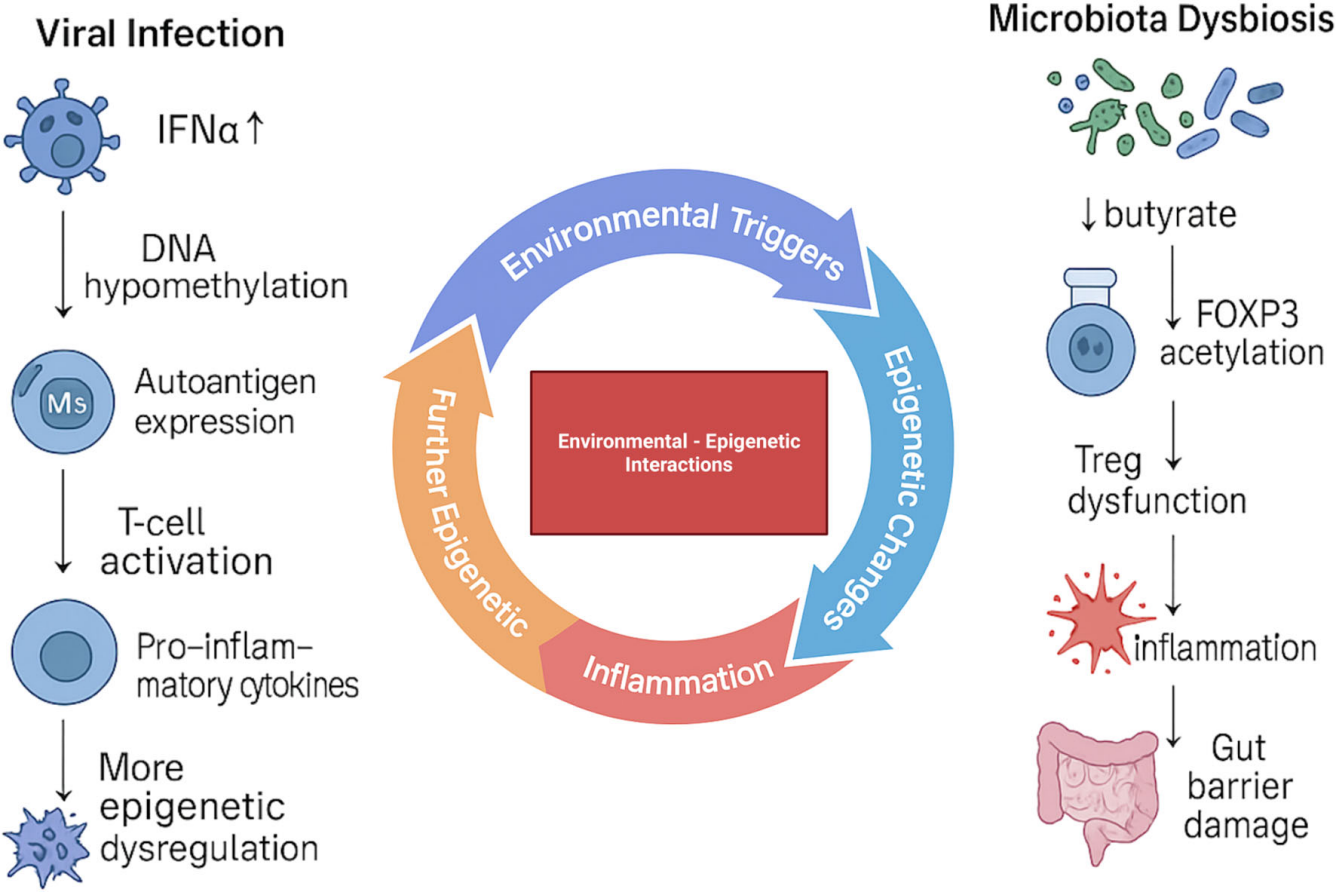
Environmental triggers and their epigenetic impact on T1D pathogenesis. Viral infections promote DNA hypomethylation, autoantigen expression, T-cell activation, and pro-inflammatory cytokine release, leading to progressive epigenetic dysregulation. In parallel, microbiota dysbiosis reduces butyrate availability, decreases FOXP3 acetylation, and impairs Treg function, driving inflammation and gut barrier damage. Together, these environmental–epigenetic interactions amplify immune dysregulation and contribute to the development of T1D.

It is currently unclear what causes pancreatic β cell loss and the development of T1D in certain people, despite the distinct roles of environmental risk factors and genetic vulnerability. There is a growing theory that the pathophysiology of T1D is significantly influenced by the interplay between genetic predisposition and environmental variables (**Figure 3**). The impact of gene variations that cause autoimmunity and result in T1D clinical symptoms may be amplified by environmental variables (59).

Together with environmental variables, epigenetic modulators have become important regulators of gene expression and cellular phenotype (34, 132–136). One of the main molecular pathways through which gene-environment interactions may heighten vulnerability to T1D is thought to be epigenetics (12, 133, 137). Investigations into epigenetic mechanisms, such as changes in DNA methylation, have revealed abnormal patterns in genes related to insulin control and immune function in people with T1D (138–141) Laajala et al. did not observe differences in DNA methylation between cases and controls in cord blood samples (142). By contrast, Johnson et al. reported DNA methylation changes that preceded seroconversion, indicating that methylation alterations can occur before the appearance of islet autoantibodies. They analyzed multiple pre-disease peripheral-blood samples and identified longitudinal differences in the rate of age-related methylation change at 10 genomic regions. Several of these differences were detectable as early as birth and in samples taken before onset of islet autoimmunity (143). Additionally, in the setting of T1D, histone alterations have demonstrated their impact on immune response gene dysregulation (144). Another aspect of epigenetics that has been highlighted is the function of miRNAs, specifically in regulating inflammatory and immunological responses in T1D (133, 145–148). In addition to their implications for biomarker discovery, epigenetic changes linked to T1D risk also pave the way for precision medicine approaches in T1D diagnosis, risk assessment, and treatment (59).

## 5 Epigenetic biomarkers and therapeutic potential

Epigenetic biomarkers are emerging as valuable tools that can be used in understanding, diagnosing, and potentially treating various diseases including T1D. Longitudinal studies show that specific methylation changes occur before clinical disease onset. In T1D, early demethylation events at immune and β-cell genes can be detected months to years before diagnosis (20). For instance, hypomethylation at the INS promoter, a hallmark of active insulin transcription, correlates with β-cell function and can be detected in circulating cell-free DNA (15, 17, 29).

Similarly, circulating miRNA pose as a promising biomarker and predictor of T1D progression. miR-25 has been found to be negatively associated with residual β-cell function, and positively associated with glycemic control 3 months after onset (149). This suggests that miR-25 may have a role in cell proliferation of pancreatic endocrine cells, thus making it of benefit in evaluating T1D progression and management. Another study suggested the usage of hsa-miR-1-3p in monitoring T1D progression and associated cardiovascular complications (146). Assessing for such epigenetic changes may aid in early detection, diagnosis, and prognosis of T1D among other diseases as well.

Epigenetic mechanisms are being explored as therapeutic targets. One promising strategy involves the use of small molecule inhibitors that target enzymes involved in epigenetic modifications including HDACs and DNMTs. HDAC inhibitors can promote a more permissive chromatin state, thereby enhancing the expression of genes involved in immune regulation and tolerance (150, 151). DNMT inhibitors, on the other hand, may reverse aberrant DNA hypermethylation and restore the expression of silenced checkpoint inhibitors such as PD-1 or CTLA-4 (152). Early preclinical models suggest that modulating these enzymes in T-cells may reduce autoreactivity and promote immune tolerance in autoimmune settings, although translation to humans is still in early stages (153).

Another emerging area of therapeutic research involves miRNA-based therapies. Since specific miRNAs contribute to the dysregulation of T-cell function in T1D, strategies that restore the balance of miRNA expression may help re-establish immune homeostasis (154). This could involve the use of miRNA mimics to restore deficient regulatory miRNAs or antagomirs to inhibit pro-inflammatory miRNAs. While these approaches offer a degree of precision not seen with conventional immunosuppressive therapies and may reduce off-target effects, challenges remain regarding delivery methods, tissue specificity, and potential immune responses. CRISPR/dCas9-mediated epigenetic editing is an emerging approach that enables precise, reversible control of gene expression without altering the DNA sequence. Unlike conventional CRISPR, the catalytically inactive “dead” Cas9 (dCas9) is fused to epigenetic modifiers such as p300 (a histone acetyltransferase) or TET1 (a DNA demethylase) and guided to specific genomic loci by custom-designed guide RNAs. This system allows researchers to modulate the epigenetic landscape of immune-regulatory genes in T-cells with high specificity. For example, targeting dCas9-p300 to the promoter of the FOXP3 gene in mouse primary T-cells significantly enhanced and stabilized FOXP3 expression, promoting a regulatory T-cell phenotype even under inflammatory conditions (155). Similarly, in human T-cell models, dCas9-TET1 systems have been used to reduce methylation at FOXP3 enhancer regions and induce functional suppressive Treg-like cells (156). These findings highlight the potential of epigenetic editing to reprogram autoreactive T-cells, restore immune tolerance, and ultimately serve as a targeted, gene-specific immunotherapy for autoimmune diseases such as T1D; however, clinical translation is limited, and challenges such as efficient *in vivo* delivery, immune cell targeting, and off-target effects remain to be addressed.

## 6 Conclusion

The immunological landscape of T1D is significantly shaped by epigenetic mechanisms, particularly in regulating T-cell growth, activation, and tolerance. These pathways operate at the intersection of genetic susceptibility and environmental exposures, offering a more integrated view of T1D pathogenesis. Recent findings on DNA methylation, histone remodeling, and non-coding RNAs shed light on why immune tolerance fails in some individuals but not others. Crucially, the reversibility of epigenetic modifications enables the possibility of therapeutic immune cell reprogramming. At the same time, epigenetic signatures hold promise as biomarkers for early risk stratification, prediction of disease progression, and monitoring of therapeutic response. Despite this potential, major challenges remain, including limited understanding of the causal hierarchy among epigenetic changes, variability across patient populations, and difficulty distinguishing disease-driving modifications from secondary changes. As tools such as CRISPR-based editing and single-cell epigenomics advance, integrating biomarker discovery with mechanistic insights will be essential for translating epigenetic research into durable and precise strategies for preventing or delaying T1D onset.
